# Higher-order error bound for the difference of two functions

**DOI:** 10.1186/s13660-018-1883-8

**Published:** 2018-10-23

**Authors:** Hui Huang, Mengxue Xia

**Affiliations:** Department of Mathematics, Yunnan University, Kunming, P.R. China

**Keywords:** 49J52, 90C31, Error bound, Difference function, Ekeland variational principle

## Abstract

Error bounds play an important role in the research of mathematical programming. Using some techniques of nonsmooth analysis, we establish some results on the existence of higher-order error bounds for difference functions with set constraints.

## Introduction

Let *X* be a Banach space, and let *Ω* be a nonempty closed convex subset of *X*. Let $f: X\rightarrow\mathbb{R}\cup\{+\infty\}$ be a proper lower semicontinuous function. We assume that

$$S=\bigl\{ x\in\varOmega| f(x)\leq0\bigr\} \neq\emptyset. $$

Let $a\in S$, $\tau>0$, and $\lambda>0$. We say that *f* has a *λ*-order local error bound *τ* at *a* if there exists $\delta>0$ such that

1.1 $$ d(x,S)^{\lambda}\leq\bigl[f(x)\bigr]_{+}, \quad \forall x\in a+\delta B_{X}, $$

where $B_{X}$ denotes the closed unit ball of *X*, $d(x,S)=\inf\{\| x-y\| | y\in S\}$, and $[f(x)]_{+}=\max\{f(x), 0\}$. We say that *f* has a *λ*-order global error bound *τ* if $a+\delta B_{X}$ in (1.1) can be replaced by the whole space *X*.

Error bounds play an important role in convergence and perturbation analysis of some algorithms and mathematical programming [1–3]. In the last twenty years, many researchers studied error bounds and obtained a lot of interesting results; see the survey papers [2, 3] and the references therein. However, these results are mainly concerned with error bounds in the case $\lambda=1$. Recently, Huang [4] considered higher-order error bounds for strongly convex multifunctions. Huang [5] and Huang and Li [6] also considered mixed-order error bounds for gamma paraconvex multifunctions. Zheng and Ng [7] studied Hölder weak sharp minimizers, which closely relate to error bounds for lower semicontinuous functions.

Recall that a function $f: X\rightarrow\mathbb{R}\cup\{+\infty\}$ is said to be a difference (DC) function if *f* is the difference of two (convex) functions. Many nonconvex optimization problems are difference structure optimization problems. In 2012, Le Thi, Pham Dinh, and Ngai [8] studied error bounds for DC functions in $\mathbb{R}^{n}$. In 2014, Huang and Li [9] studied error bounds for DC multifunctions. In 2016, Van Hang and Yao [10] established sufficient conditions for the existence of error bounds for difference functions and applications. However, all results mentioned in this paragraph are concerned with $\lambda =1$. It is natural for us to consider higher-order error bounds for difference functions.

The rest of this paper is organized as follows. In Sect. 2, we give some notions. In Sect. 3, we establish sufficient and necessary conditions for the existence of higher-order error bounds for difference functions with set constraints in terms of nonsmooth analysis tools.

## Preliminaries

Let *X* be a real Banach space, and let $\tau, \lambda>0$. We say that $f: X\rightarrow \mathbb{R}\cup\{+\infty\}$ is a *λ*-order strongly convex function with modulus *τ* if for all $x, y\in X$ and $t\in(0,1)$,

$$\tau t(1-t) \Vert x-y \Vert ^{\lambda}+f\bigl(tx+(1-t)y\bigr)\leq tf(x)+(1-t)f(y). $$

In the particular case $\tau=0$, strongly convex functions reduce to convex functions. We say that *f* is proper if its domain $\operatorname{dom}(f):=\{x\in X | f(x)<+\infty\}\neq\emptyset$. Let $x_{0}\in\operatorname{dom}(f)$. We say that *f* is lower semicontinuous at $x_{0}$ if

$$\liminf _{x\rightarrow x_{0}}f(x)\geq f(x_{0}). $$

Let $u\in X$. We define

$$\underline{f}'(x_{0};u)=\liminf _{t\rightarrow0^{+}}\frac {f(x_{0}+tu)-f(x_{0})}{t}. $$

It is known that

$$\underline{f}'(x_{0}; u)=f'(x_{0}; u)=\lim _{t\rightarrow 0^{+}}\frac{f(x_{0}+tu)-f(x_{0})}{t} $$

if *f* is a convex function. For a convex function *f* and $\bar{x}\in \operatorname{dom}(f)$, the subdifferential of *f* at *x̄* is defined as

$$\partial f(\bar{x})=\bigl\{ x^{*}\in X^{*} | \bigl\langle x^{*}, x-\bar {x}\bigr\rangle \leq f(x)-f(\bar{x}), \forall x\in X\bigr\} . $$

Let *Ω* be a closed convex subset of *X*, and let $a\in\varOmega$. The tangent cone of *Ω* at *a* is defined as

$$T(\varOmega,a)=\bigl\{ v\in X | \exists t_{n}>0, t_{n} \rightarrow0^{+}, v_{n}\in X, v_{n}\rightarrow v \mbox{ such that } a+t_{n}v_{n}\in\varOmega \bigr\} . $$

Let $A\subset\mathbb{R}^{n}$ and $x\in\mathbb{R}^{n}$. The projection of *x* on *A* is defined as

$$P_{A}(x)=\bigl\{ y\in A | \Vert x-y \Vert =d(x,A)\bigr\} . $$

The limiting normal cone of *A* at $a\in A$ [11] is defined as

$$\begin{aligned} N(A;a)={}&\bigl\{ v\in\mathbb{R}^{n} | \exists x_{k}\in\mathbb{R}^{n}, x_{k}\rightarrow a, t_{k}>0, u_{k}\in P_{A}(x_{k}) \\ &\mbox{ such that } t_{k}(x_{k}-u_{k}) \rightarrow v\bigr\} . \end{aligned} $$

Let $\bar{x}\in X$ and $\delta>0$. By $B(\bar{x}, \delta)$ we denote the open ball with center at *x̄* and radius *δ* and by $S_{X}$ the unit sphere of *X*. By $\operatorname{bdry}(S)$ we denote the boundary of a set *S*.

## Main results

In this section, we assume that *X* is a real Banach space unless stated otherwise, $\varOmega\subset X$ is a nonempty closed convex set, $g, h: X\rightarrow\mathbb{R}\cup\{+\infty\}$ are two proper functions, and $S=\{x\in\varOmega | g(x)-h(x)\leq 0\}\neq\emptyset$. We first establish sufficient conditions for the existence of higher-order error bounds for difference functions.

### Theorem 3.1

*Let* $g: X\rightarrow\mathbb{R}\cup\{ +\infty\}$ *be a lower semicontinuous function*, *and let* $h: X\rightarrow\mathbb{R}$ *be a continuous function*. *Let* $\tau>0$, $\delta>0$, $\lambda>0$, *and* $\bar{x}\in S$. *Suppose that*, *for each* $x\in\varOmega\setminus S$ *such that* $\|x-\bar{x}\|<\delta$, *there exists* $u\in T(\varOmega; x)\cap S_{X}$ *such that*

3.1 $$ \underline{g}'(x; u)+\tau d(x, S)^{\frac{1}{\lambda}}\leq \underline {h}'(x; u). $$

*Then*

3.2 $$ \frac{\tau}{4\cdot2^{\frac{1}{\lambda}}} d(x, S)^{\frac{1+\lambda }{\lambda}}\leq\bigl[g(x)-h(x) \bigr]_{+}, \quad \forall x\in\varOmega\cap B\biggl(\bar{x}, \frac{2}{3}\delta\biggr). $$

### Proof

Suppose on the contrary that (3.2) is not true. Then there exists $\tilde{x}\in\varOmega$ such that $\|\tilde{x}-\bar{x}\|<\frac{2}{3}\delta$ and

3.3 $$ \bigl[g(\tilde{x})-h(\tilde{x})\bigr]_{+}< \frac{\tau}{4\cdot2^{\frac{1}{\lambda }}} d(\tilde{x},S)^{\frac{1+\lambda}{\lambda}}. $$

Since $\inf_{x\in\varOmega} [g(x)-h(x)]_{+}=0$, it follows from (3.3) that

$$\bigl[g(\tilde{x})-h(\tilde{x})\bigr]_{+}< \inf _{x\in\varOmega} \bigl[g(x)-h(x)\bigr]_{+}+\frac{\tau}{4\cdot2^{\frac{1}{\lambda}}} d(\tilde {x},S)^{\frac{1+\lambda}{\lambda}}. $$

By the completeness of *X* and the closedness of *Ω* we know that *Ω* is a complete metric space with respect to the metric induced by the norm of *X*. By the Ekeland variational principle [12] there exists $x_{0}\in\varOmega$ such that

3.4 $$ \Vert x_{0}-\tilde{x} \Vert \leq\frac{1}{2} d( \tilde{x},S) $$

and

3.5 $$ \bigl[g(x_{0})-h(x_{0})\bigr]_{+}< \bigl[g(x)-h(x)\bigr]_{+}+\frac{\tau}{2\cdot2^{\frac {1}{\lambda}}} d(\tilde{x},S)^{\frac{1}{\lambda}} \Vert x-x_{0} \Vert , \quad \forall x\in\varOmega\setminus \{x_{0}\}. $$

From (3.4) we have that $x_{0}\notin S$, and so $g(x_{0})-h(x_{0})>0$. Let $u\in T(\varOmega; x_{0})\cap S_{X}$. Since *Ω* is a convex set, there exists $t_{0}>0$ such that $x_{0}+tu\in\varOmega$ for all $t\in(0, t_{0})$. Since $g-h$ is lower semicontinuous at $x_{0}$, there exists $t_{1}\in(0, t_{0})$ such that

$$\bigl[g(x_{0}+tu)-h(x_{0}+tu)\bigr]_{+}=g(x_{0}+tu)-h(x_{0}+tu) $$

for all $t\in(0, t_{1})$. It follows from (3.5) that

$$g(x_{0})-h(x_{0})< g(x_{0}+tu)-h(x_{0}+tu)+ \frac{\tau}{2\cdot2^{\frac {1}{\lambda}}} d(\tilde{x},S)^{\frac{1}{\lambda}} \Vert x_{0}+tu-x_{0} \Vert $$

for all $t\in(0, t_{1})$, that is,

$$h(x_{0}+tu)-h(x_{0})< g(x_{0}+tu)-g(x_{0})+ \frac{\tau}{2\cdot2^{\frac {1}{\lambda}}} d(\tilde{x},S)^{\frac{1}{\lambda}}t $$

since $\|u\|=1$. Therefore

$$\frac{h(x_{0}+tu)-h(x_{0})}{t}< \frac{g(x_{0}+tu)-g(x_{0})}{t}+\frac{\tau }{2\cdot2^{\frac{1}{\lambda}}}d(\tilde{x},S)^{\frac{1}{\lambda}}. $$

Taking lim inf as $t\rightarrow0^{+}$, we get

3.6 $$ \underline{h}'(x_{0};u)\leq \underline{g}'(x_{0};u)+\frac{\tau}{2\cdot 2^{\frac{1}{\lambda}}} d( \tilde{x},S)^{\frac{1}{\lambda}}. $$

By (3.4) we have

$$d(\tilde{x},S)\leq d(x_{0},S)+ \Vert \tilde{x}-x_{0} \Vert \leq d(x_{0},S)+\frac {1}{2}d(\tilde{x},S), $$

and so $d(\tilde{x},S)\leq2d(x_{0},S)$. This inequality and (3.6) imply that

3.7 $$ \underline{h}'(x_{0};u)\leq \underline{g}'(x_{0}; u)+\frac{\tau}{2\cdot 2^{\frac{1}{\lambda}}}2^{\frac{1}{\lambda}}d(x_{0},S)^{\frac{1}{\lambda}} =\underline{g}'(x_{0}; u)+\frac{\tau}{2}d(x_{0},S)^{\frac{1}{\lambda}}. $$

By (3.4) we get

3.8 $$ \begin{aligned} \Vert x_{0}-\bar{x} \Vert &\leq \Vert x_{0}-\tilde{x} \Vert + \Vert \tilde{x}- \bar{x} \Vert \leq \frac{1}{2}d(\tilde{x},S)+ \Vert \tilde{x}-\bar{x} \Vert \\ &\leq\frac{1}{2} \Vert \tilde{x}-\bar{x} \Vert + \Vert \tilde{x}- \bar{x} \Vert < \delta. \end{aligned} $$

Inequalities (3.7) and (3.8) are a contradiction to (3.1). The proof is completed. □

We now give necessary conditions for the existence of higher-order error bounds for difference functions.

### Theorem 3.2

*Let* $g, h: X\rightarrow\mathbb{R}$ *be two continuous convex functions*. *Let* $\tau>0$, $\delta>0$, $\lambda >0$, *and* $\bar{x}\in S$. *Suppose that*

3.9 $$ \tau d(x, S)^{\frac{1+\lambda}{\lambda}}\leq\bigl[g(x)-h(x)\bigr]_{+}, \quad \forall x\in\varOmega\cap B(\bar{x},\delta). $$

*Then*, *for each* $x\in\varOmega\setminus S$ *such that* $\|x-\bar{x}\|<\frac {\delta}{2}$, *there exist* $a\in S$ *and* $u\in T(\varOmega; x)\cap S_{X}$ *such that*

3.10 $$ g'(x; u)+\frac{\tau}{2} d(x, S)^{\frac{1}{\lambda}}\leq h'(a; u). $$

### Proof

Let $x\in\varOmega\setminus S$ be such that $\|x-\bar{x}\| <\frac{\delta}{2}$. Take $a\in\operatorname{bdry}(S)$ such that$\|x-a\| <2d(x,S)$. Then

$$\Vert x-a \Vert < 2d(x,S)\leq2 \Vert x-\bar{x} \Vert < \delta. $$

As $a\in\operatorname{bdry}(S)$, we have $g(a)-h(a)=0$. By (3.9),

$$\frac{\tau}{2}d(x,S)^{\frac{1}{\lambda}} \Vert x-a \Vert \leq \tau d(x, S)^{\frac {1+\lambda}{\lambda}}\leq g(x)-h(x)-\bigl[g(a)-h(a)\bigr], $$

that is,

3.11 $$ h(x)-h(a)+\frac{\tau}{2} d(x,S)^{\frac{1}{\lambda}} \Vert x-a \Vert \leq g(x)-g(a). $$

Let $x^{*}\in\partial g(x)$ and $a^{*}\in\partial h(a)$. Then

$$\bigl\langle x^{*},a-x\bigr\rangle \leq g(a)-g(x), \qquad \bigl\langle a^{*},x-a\bigr\rangle \leq h(x)-h(a). $$

It follows from (3.11) that

$$\biggl\langle a^{*}, \frac{x-a}{ \Vert x-a \Vert }\biggr\rangle + \frac{\tau}{2} d(x,S)^{\frac {1}{\lambda}}\leq \biggl\langle x^{*}, \frac{x-a}{ \Vert x-a \Vert }\biggr\rangle . $$

Denote $u:=\frac{a-x}{\|a-x\|}$. Then $u\in T(\varOmega; x)\cap S_{X}$ since *Ω* is a convex set. The last inequality implies that

$$-\max _{a^{*}\in\partial h(a)}\bigl\langle a^{*},u\bigr\rangle + \frac{\tau }{2} d(x,S)^{\frac{1}{\lambda}}\leq-\max _{x^{*}\in\partial g(x)}\bigl\langle x^{*},u \bigr\rangle . $$

Since $h'(a; u)=\max_{a^{*}\in\partial h(a)} \langle a^{*}, u\rangle$ and $g'(x; u)=\max_{x^{*}\in\partial g(x)} \langle x^{*}, u\rangle $, from this inequality it follows that

$$-h'(a;u)+\frac{\tau}{2} d(x,S)^{\frac{1}{\lambda}} \leq-g'(x;u). $$

Therefore (3.10) is verified. □

### Corollary 3.1

*Let* $g: X\rightarrow\mathbb{R}$ *be a continuous convex function*, *and let* $\lambda>0$. *Then the following two statements are equivalent*:

(i) *There exist* $\tau>0$ *and* $\delta>0$ *such that*
  $$\tau d(x,S)^{\frac{1+\lambda}{\lambda}}\leq\bigl[g(x)\bigr]_{+}, \quad \forall x \in\varOmega\cap B(\bar{x}, \delta); $$
(ii) *There exist* $\tau'>0$ *and* $\delta'>0$ *such that*, *for each* $x\in\varOmega\setminus S$ *with* $\|x-\bar{x}\|<\delta'$, *there exists* $u\in T(\varOmega; x)\cap S_{X}$ *such that*
  $$g'(x;u)\leq-\tau' d(x,S)^{\frac{1}{\lambda}}. $$

### Proof

The conclusion directly follows from Theorems 3.1 and 3.2 by taking $h=0$. □

### Theorem 3.3

*Let* $g: X\rightarrow\mathbb{R}$ *be a* *λ*-*order strongly convex function with modulus τ*, *and let* $h: X\rightarrow \mathbb{R}$ *be a convex function*. *If for each* $x\in\varOmega\setminus S$, *there exists* $y\in\operatorname{bdry}(S)$ *such that*

3.12 $$ g'(y;x-y)+h'(x;y-x)\geq0, $$

*then*

$$\tau d(x,S)^{\lambda}\leq\bigl[g(x)-h(x)\bigr]_{+}, \quad \forall x\in\varOmega. $$

### Proof

Let $x\in\varOmega$. Without loss of generality, we may assume that $x\in\varOmega\setminus S$. Take $y\in\operatorname{bdry}(S)$ such that (3.12) holds. Let $t\in(0, 1)$. Since *g* is a *λ*-order strongly convex function with modulus *η*, we have

$$\tau t(1-t) \Vert x-y \Vert ^{\lambda}\leq tg(x)+(1-t)g(y)-g \bigl(tx+(1-t)y\bigr), $$

that is,

$$\tau(1-t) \Vert x-y \Vert ^{\lambda}\leq g(x)-g(y)-\frac{g(y+t(x-y))-g(y)}{t}. $$

Letting $t\rightarrow0^{+}$, we get

3.13 $$ \tau \Vert x-y \Vert ^{\lambda}\leq g(x)-g(y)-g'(y;x-y). $$

Since *h* is a convex function, we have

3.14 $$ 0\leq h(y)-h(x)-h'(x;y-x). $$

Adding (3.13) and (3.14), we have

3.15 $$\begin{aligned} \tau \Vert x-y \Vert ^{\lambda} \leq& g(x)-h(x)- \bigl[g(y)-h(y)\bigr]-\bigl[g'(y;x-y)+h'(x;y-x)\bigr] \\ \leq& g(x)-h(x), \end{aligned}$$

due to assumption (3.12) and the equality $g(y)-h(y)=0$. Since $y\in\operatorname{bdry}(S)$, it follows from (3.15) that

$$\tau d(x,S)^{\lambda}\leq g(x)-h(x)=\bigl[g(x)-h(x)\bigr]_{+}. $$

 □

We now give an example to illustrate Theorem 3.3.

### Example 3.1

Let $g, h: \mathbb{R}\rightarrow\mathbb {R}$ be defined as

$$g(x)=x^{2}, \qquad h(x)=x, \quad \forall x\in\mathbb{R}. $$

Clearly, *g* is a second-order strongly convex function with modulus $\tau=1$. It is easy to calculate that $S=\{x\in\mathbb{R} | g(x)-h(x)\leq0\}=[0,1]$. Take $\varOmega=(-\infty, 1]$. Let $x\in\varOmega\setminus S=(-\infty, 0)$. There exists $y=0\in\operatorname{bdry}(S)$ such that

$$g'(y;x-y)+h'(x;y-x)=-x>0. $$

All conditions of Theorem 3.3 are satisfied. By Theorem 3.3 we have

$$d(x,S)^{2}\leq\bigl[g(x)-h(x)\bigr]_{+}, \quad \forall x \in\varOmega. $$

### Theorem 3.4

*Take* $X=\mathbb{R}^{n}$, *and let* $f:\mathbb{R}^{n}\rightarrow\mathbb{R}$ *be an* *mth*-*order smooth function* (*m* *is a positive integer number*), $\delta>0$, $S:=\{x\in\mathbb{R}^{n} | f(x)\leq0\}\neq\emptyset$, *and* $\bar {x}\in\operatorname{bdry}(S)$. *Suppose that*, *for each* $x\in B(\bar{x},\delta)\setminus S$, *there exists* $u\in P_{S}(x)$ *such that*

$$f^{(p)}(u) (x-u)^{p}\geq0, \quad p=1, 2, \ldots, m-1, $$

*and*

3.16 $$ f^{(m)}(\bar{x}) \bigl(v^{m}\bigr)>0, \quad \forall v\in N(S, \bar{x})\setminus \{0\}. $$

*Then there exist* *τ*> *and* $\eta>0$ *such that*

$$\tau d(x,S)^{m}\leq\bigl[f(x)\bigr]_{+}, \quad \forall x\in B(\bar{x}, \eta). $$

### Proof

Suppose on the contrary that there exists a sequence $\{ x_{k}\}\subset B(\bar{x},\delta)$ with $x_{k}\rightarrow\bar{x}$ such that

3.17 $$ \frac{d(x_{k}, S)^{m}}{k}>\bigl[f(x_{k})\bigr]_{+}. $$

Clearly, $x_{k}\notin S$ for all *k*. By the assumption we can take $u_{k}\in P_{S}(x_{k})$ such that

3.18 $$ f^{(p)}(u_{k}) (x_{k}-u_{k})^{p} \geq0, \quad p=1, 2, \ldots, m-1. $$

Clearly, $\|x_{k}-u_{k}\|=d(x_{k},S)$. Letting $k\rightarrow\infty$, we have $u_{k}\rightarrow\bar{x}$. By (3.17) we get

3.19 $$ \frac{ \Vert x_{k}-u_{k} \Vert ^{m}}{k}>f(x_{k})-f(u_{k}) $$

since $f(u_{k})=0$. By the Taylor theorem,

3.20 $$ \begin{aligned}[b] f(x_{k})-f(u_{k})={}&f'(u_{k}) (x_{k}-u_{k})+\frac {1}{2!}f''(u_{k}) (x_{k}-u_{k})^{2}+\cdots \\ &{}+\frac{f^{(m)}(\theta_{k}x_{k}+(1-\theta_{k})u_{k})}{m!}(x_{k}-u_{k})^{m} \end{aligned} $$

for some $\theta_{k}\in(0, 1)$, and (3.18)–(3.20) imply that

$$\begin{aligned} \frac{ \Vert x_{k}-u_{k} \Vert ^{m}}{k}\geq{}& f'(u_{k}) (x_{k}-u_{k})+\frac {1}{2!}f''(u_{k}) (x_{k}-u_{k})^{2}+\cdots \\ &{}+\frac{f^{(m)}(\theta_{k}x_{k}+(1-\theta _{k})u_{k})}{m!}(x_{k}-u_{k})^{m} \\ \geq{}&\frac{f^{(m)}(\theta_{k}x_{k}+(1-\theta_{k})u_{k})}{m!}(x_{k}-u_{k})^{m}, \end{aligned} $$

that is,

3.21 $$ \frac{m!}{k}\geq f^{(m)}\bigl(\theta_{k}x_{k}+(1- \theta_{k})u_{k}\bigr) \biggl(\frac {x_{k}-u_{k}}{ \Vert x_{k}-u_{k} \Vert } \biggr)^{m}. $$

Since $u_{k}\in P_{S}(x_{k})$ and $\{\frac{x_{k}-u_{k}}{\|x_{k}-u_{k}\| }\}$ is bounded, without loss of generality, we may assume that $\frac{x_{k}-u_{k}}{\|x_{k}-u_{k}\|}\rightarrow v\in N(S, \bar{x})$. Letting $k\rightarrow\infty$ in (3.21), we have

$$f^{(m)}(\bar{x}) \bigl(v^{m}\bigr)\leq0. $$

This contradicts (3.16). The proof is completed. □

We now give an example to illustrate Theorem 3.4.

### Example 3.2

Let $f: \mathbb{R}^{2}\rightarrow\mathbb {R}$ be defined by

$$f(\xi_{1}, \xi_{2})=\xi_{1}^{2}+ \xi_{2}^{2}, \quad \forall x=(\xi _{1}, \xi_{2})\in\mathbb{R}^{2}. $$

Clearly, $S=\{(0,0)\}$. Take $\bar{x}=(0,0)\in\operatorname{bdry}(S)$. Then $N(S,\bar{x})=\mathbb{R}^{2}$. Let $x\in\mathbb{R}^{2}\setminus S$. There exists $u=(0,0)\in P_{S}(x)$ such that $f'(u)(x-u)=0$ and

$$f''(\bar{x}) \bigl(v^{2}\bigr)=2 \bigl(v_{1}^{2}+v_{2}^{2}\bigr)>0, \quad \forall v=(v_{1}, v_{2})\in N(S,\bar{x})\setminus\{0 \}. $$

All conditions of Theorem 3.4 are satisfied. By Theorem 3.4 there exists $\tau>0$ ($\tau=1$) such that

$$\tau d(x,S)^{2}\leq\bigl[f(x)\bigr]_{+}, \quad \forall x\in \mathbb{R}^{2}. $$

## Conclusion

In this paper, we establish two existence theorems of higher-order error bounds for difference functions and an existence theorem of higher-order error bounds for *m*th-order smooth functions. Moreover, the coefficients in Theorems 3.1–3.4 can be calculated. It is interesting for us to consider higher-order error bounds for DC multifunctions.

## References

[1] Hoffman A.J. (1952). On approximate solutions of systems of linear inequalities. J. Res. Natl. Bur. Stand..

[2] Azé D. (2003). A survey on error bounds for lower semicontinuous functions. Proceedings of MODE-SMAI Conference. ESAIM Proc..

[3] Pang J.S. (1997). Error bounds in mathematical programming. Math. Program., Ser. B.

[4] Huang H. (2010). Global error bounds with exponents for multifunctions with set constraints. Commun. Contemp. Math..

[5] Huang H. (2012). Coderivative conditions for error bounds of gamma-paraconvex multifunctions. Set-Valued Var. Anal..

[6] Huang H., Li R.X. (2011). Global error bounds for gamma-multifunctions. Set-Valued Var. Anal..

[7] Zheng X.Y., Ng K.F. (2015). Hölder weak sharp minimizers and Hölder tilt-stability. Nonlinear Anal. Theory Methods Appl..

[8] Le Thi H.A., Pham Dinh T., Ngai H.V. (2012). Exact penalty and error bounds in DC programming. J. Glob. Optim..

[9] Huang H., Li R.X. (2014). Error bounds for the difference of two convex multifunctions. Set-Valued Var. Anal..

[10] Van Hang N.Y., Yao J.C. (2016). Sufficient conditions for error bounds of difference functions and applications. J. Glob. Optim..

[11] Rockafellar R.T., Wets R.J.-B. (1998). Variational Analysis.

[12] Ekeland I. (1974). On the variational principle. J. Math. Anal. Appl..

